# Post-electroconvulsive therapy cognitive alterations

**DOI:** 10.1192/j.eurpsy.2023.2168

**Published:** 2023-07-19

**Authors:** K. Douk, I. Belabess, S. Belbachir

**Affiliations:** 1 Psychiatry, Military hospital Mohammed V, Rabat; 2 Psychiatric hospital Ar-Razi; 3 Psychiatry, Psychatric hospital Ar-Razi, Salé, Morocco

## Abstract

**Introduction:**

ectroconvulsive therapy (ECT) is a medical treatment most often used for patients with severe major depression or bipolar disorder who have not responded to other treatments, as well as for resistant schizophrenia, and consists of brief electrical stimulation of the brain while the patient is under anesthesia. It is usually administered by a team of trained health care professionals, including a psychiatrist, an anesthesiologist and a nurse or physician assistant.

The mode of action is not well known, but it is assumed that ECT is associated with a significant reduction in brain connections in the dorsolateral prefrontal cortex, which is associated with a significant decrease in depressive symptoms. This would support the hypothesis that hyper-connectivity in this area of the brain is closely linked to depression.

Despite its great effectiveness, this technique remains limited by numerous contraindications and exposes the patient to a multitude of short-, medium- and long-term side effects, notably cognitive disorders.

**Objectives:**

To shed light on the cognitive disorders affecting mnesic processes, learning and thinking after electroconvulsive therapy.

**Methods:**

We have performed a systematic literature review using the following keywords on the GoogleScholar database: cognitive impairment post electroconvulsive therapy, cognitive effects of electroconvulsive therapy, electroconvulsive side effects, ECT.

**Results:**

Most of the studies on cognitive disorders after ECT have focused on mnesic abilities, in particular the deterioration of anterograde memory which is at its maximum just after the session and which recovers progressively and spontaneously. The deterioration of retrograde memory, on the other hand, depends on the administered dose, the type and the location of the electrodes, and has a greater tendency to affect recent memories than old ones.

Other studies have focused on the speed of the process of information, which is also affected, with a significant decrease, especially in depressed people, a decrease that can be progressively resolved with time. Concerning attentiveness, some studies have noted a minimal decrease, especially in the lateral visual fields, and a perseveration in verbal expression. These studies also noted a deterioration of executive functions with a decrease in performance on the STROOP and MTM tests and also in verbal fluency.

**Conclusions:**

The occurrence and preservation of cognitive deficits after ECT is a much debated subject with many controversial studies, and despite the numerous studies on this subject, we are still far from conclusive and exploitable results, especially with the intricacy of the diversity of parameters, materials and techniques of ECT, and also because of other factors such as individual peculiarities, neurological co-morbidities, and polymedication to psychotropic drugs.

**Disclosure of Interest:**

None Declared

